# Workplace Bullying and Harassment in Higher Education Institutions: A Scoping Review

**DOI:** 10.3390/ijerph21091173

**Published:** 2024-09-03

**Authors:** Margaret Hodgins, Rhona Kane, Yariv Itzkovich, Declan Fahie

**Affiliations:** 1 Department of Health Promotion, School of Health Sciences, University of Galway, H91TK33 Galway, Ireland; 2 Department of Human Resource Management, Faculty of Social Sciences and Humanities, Kinneret College on the Sea of Galilee, Zemach Emek HaYarden Mobile Post 15132, Israel; 3 School of Education, University College of Dublin, D04EW81 Dublin, Ireland

**Keywords:** workplace bullying, harassment, higher education institution, neoliberalism, gendered power dynamics, precarity

## Abstract

Workplace bullying is broadly defined as a detrimental form of negative micro-political interaction(s) incorporating a range of aggressive interpersonal behaviours. While targeted toxic behaviour based upon legally protected grounds such as ethnicity, gender, or sexual orientation is conceptualised as harassment, this paper positions harassment as a constituent subset of workplace bullying—distinct, but inextricably linked to the broader landscape of workplace predation and incivility. Meta-analyses of cross-sectional and longitudinal studies demonstrate a robust relationship between being bullied and compromised health, and some sectors, e.g., education, display higher than average levels of exposure, suggesting that contexts matter. The higher education sector is the focus of this scoping review. High rates of bullying have been reported in Higher Education Institutions (HEIs), where many of the organisational factors that drive bullying are present. One systematic literature review has been carried out on bullying in HEIs, reviewing papers prior to 2013. Since the sector has seen considerable contextual change since that time, another review is timely. This systematic scoping review aims to identify the volume, range, nature, and characteristics of studies of workplace bullying in HEIs between 2003 and 2023, with a specific focus on how the *context* of HEIs contributes to the enactment and/or the response to workplace bullying. To this end, 3179 records were identified, with 140 papers charted to identify methods, institution, population, and country. Forty-seven papers were subjected to full-text review for the exploration of contextual factors. Priorities for future research lie in addressing the pernicious effects of neoliberal governance models as well as the complex and intersecting power relations that are unique to higher education.

## 1. Introduction

Workplace bullying is an especially detrimental form of negative behaviour incorporating a range of aggressive interpersonal behaviours [1], including verbal abuse or incivility and micro-political “game playing” for personal gain [2]. It also includes more subtle enactments of punitive and unfair management practices, such as exclusion from key processes, assigning unreasonable duties, or making work tasks impossible [3,4,5,6]. Workplace bullying is a notoriously difficult and contested construct. Preventing or ameliorating it continues to present a significant challenge in organisations. It is a complex phenomenon in a complex setting.

In the absence of consensus on an exact definition of workplace bullying, there is broad agreement that ‘…the nature of the bullying situation involves persistent and systematic victimisation of a colleague or a subordinate through the repeated use of various kinds of aggressive behaviours over a long (taken to mean 3–6 months) period of time and in a situation where victims (while victim is term used in the quotation, target is the authors’ preferred term and will be used throughout the article) have difficulty in defending themselves.’ ([7], p. 41).

Bullying is presented here as a superordinate concept aligned with harassment based on being a member of a protected class, for example, bullying based on ethnicity, gender, or sexual orientation [1,8,9,10]. Early approaches to the study of sexual harassment maintained it to be conceptually distinct from bullying on the basis that it was seen to be about sex and gender conflict, whereas bullying or psychological violence was deemed gender-neutral [11]. However, evidence of overlap has been found in both the gendered nature of bullying [12] and the re-focusing of sexual harassment to include sex-based harassment as an expression of power and dominance in a variety of contexts in order to reinforce an existing gender hierarchy [13]. Workplace bullying presents as interpersonal and relational difficulties, but is rooted in organisational issues of power and control. Accepting the overlap between bullying and sexual harassment facilitates an understanding of violence being located in both societal and structural factors, presenting, or playing out, through organisational and individual factors.

The impact of workplace bullying on the health and well-being of workers is unequivocal; meta-analyses of both cross-sectional and longitudinal studies demonstrate a robust relationship between the experience of being bullied and compromised health and well-being [1,14]. Demonstrated across diverse occupations, sectors, and cultures, this is one of the most well-established findings in the workplace-bullying literature. Cross-sectional, multivariate, and longitudinal studies have demonstrated that bullying predicts reduced or poorer health, while poor health also raises the risk of exposure to bullying. Studies focusing explicitly on sexual harassment as a form of bullying also demonstrate an association with poor mental health [15,16,17].

Qualitative studies reinforce the damage of bullying and demonstrate its nuanced impact. While quantitative studies usually measure bullying at the individual level, qualitative studies expose how bullying is a social relational problem; relationships are both causes and casualties. Targets lose trust in their work group or the organisation, are disillusioned, and experience shame and/or compromised professional identities [2,18,19,20,21,22]. Qualitative studies reveal that bullying impacts self-worth, and can engender feelings of self-contempt, guilt, isolation, and vulnerability [23,24,25,26]. The words used by targets to describe the personal impact are graphic: ‘*He has left me scarred*’ ([27], p. 290); ‘*She has actually cracked my health*’ ([28], p. 113); “*I felt desperate, used, and dumped like trash*’. ([29], p. 40). This gives voice to the claim that exposure to bullying in work is a more crippling problem for employees than all other kinds of work-related stress put together [30,31].

Within the literature on the drivers of workplace bullying, two sets of factors are seen to affect workplace bullying, reflected in two strands of investigation: individual dispositional factors (such as personality traits) and work environment factors (organisational stressors). However, the action of one set of factors does not preclude interaction with the other [1]. Individual factors, such as conscientiousness, are evident, but appear to interact with work environmental factors [1,32]. Work stressors and a poor psychosocial work environment, for example, role conflict, role ambiguity, or high job demands, have been consistently identified as antecedents to bullying [32,33,34,35]. Those who leave jobs due to bullying are less likely to report bullying in new jobs and report significantly reduced anxiety [12]. Higher-order work environment factors such as workplace culture are repeatedly cited as influencing work environment factors (e.g., [36,37,38,39]). In the most general sense, for example, despite the contribution of individual and work environment factors, bullying will be present and persistent only where organisations, explicitly or implicitly, tolerate it [7,40,41]. A less studied aspect of bullying, however, is institutional or depersonalised bullying where the institution itself, through it structures and processes, is regarded as responsible for bullying practices [42,43,44]. Consistent with these findings, several conceptual frameworks or models of workplace bullying have been offered (for a comprehensive review of theoretical models see [45]). Common to all is the idea of the dynamic interplay of individual, organisational, and wider social factors.

Finally, despite evidence that bullying has a substantial negative impact, both at the aforementioned level of individual health [1,23,25,46] and organizational productivity and costs [47,48,49], organisations typically fail to prevent or ameliorate it. The evidence that workplaces are ineffectual in protecting workers from bullying is drawn from studies on turnover intention and quit rates, studies of self-reported actions taken (or not taken), qualitative accounts of targets’ experiences of organizational responses, and studies of HR perspectives on organizational responses [43,50,51]. Workers have a significant need and legitimate expectation for fairness and reasonable protection in their workplaces. When it fails to materialize, and worse, the perpetrator of psychological abuse or violence is not reprimanded or sanctioned, it exacerbates compromised health [26]. It is hard to over-state the sense of betrayal for targets.

Given the complexity of workplace bullying, and the difficulties addressing it, a more nuanced or contextual approach is required. Nielsen et al. (2018), while cautioning against comparisons without due consideration of the methodological moderators of location, instrument, and sampling strategy, arrived at an average prevalence for being bullied in the workplace of 14.6%, however with considerable variation by occupational sector [1]. The health and social care sector, the defence sector, and the educational sector display higher than average levels of exposure [4,31,35,52]. The latter, specifically higher education, is the focus of this study.

### Workplace Bullying and Harassment in Higher Education Institutions (HEIs)

While workplace bullying has a universal presence across all workplaces globally [53], places and spaces matter. Both sectoral and institutional contexts are critical to understanding how workplace toxicity manifests and how it might best be addressed. Higher Education Institutions (HEIs) are one such context in which employees appear to have high levels of exposure. Although a comparison of prevalence estimates is challenging due to variations in measurement approaches [54] and varying focuses of enquiry (e.g., academic staff, professional staff, and non-disaggregated student and staff samples), alarmingly high rates of bullying have been reported in HEIs. Sixty-five percent of the staff of six New Zealand universities reported being bullied in the previous year [55]. Meanwhile, rates of 44%, 52%, and 46% have been found, respectively, in individual universities in Canada, Ethiopia, and the UK [56,57,58]. While these estimates must be interpreted prudently given the limited information on the provenance of the instruments employed and the cut-off points for “case-ness”, they are indicative of a serious problem in the sector. A meta-analysis of studies of sexual harassment in higher education finds similarly concerning rates of ca. 25% although, as with measures of bullying, prevalence rates are influenced by a range of methodological and contextual factors and often include both staff and students [59].

Certain contextual factors signal the likelihood of higher bullying risk in HEIs. Organisational change is an established precursor for workplace bullying [41,60,61], and in this context the higher educational sector has experienced an exceptional degree of change in recent years. External factors such as globalisation and a neoliberal agenda have led to massification, managerialism, and marketisation in publicly funded HEIs [62,63]. Intersecting with economic recessions across the world and massive funding cuts, HEIs have experienced a very significant intensification of work, with reduced staffing, increased precarity [64], and changes to ways of working. Such changes include, but are not limited to, the curtailing of academic freedom [65], persistent gender inequity [66], performativity and competition [67,68], and the need to implement rapid technological change, particularly in the context of the COVID-19 pandemic.

While these contextual factors for HEIs render the high rates of bullying unsurprising, there appears to be relatively few studies on the higher educational sector. In a review of prevalence studies that identified 174 samples over a 30-year period (1989–2019), only 10 were drawn from the higher education sector [69]. An overview of studies on workplace bullying in higher education [70] lists only 15 studies in a 16-year period, while the only systematic review of harassment (specifically) in higher education identified 51 articles published between 1994 and 2013, reporting the types and measures of harassment employed, the causes and instigators of harassment, consequences and comorbidities, and the interventions employed to address harassment [71]. Bondestam and Lundquist’s (2020) systematic review of sexual harassment in higher education is comprehensive, but does not differentiate between student and staff experiences. This relatively small corpus of work is notable when compared, for example, to reviews of bullying in nursing [72] and health care [73]. Henning’s systematic review only found 5 studies that incorporated qualitative interviews, 1 wholly qualitative study, and 11 studies conducted outside the US [71]. Taking the substantial changes in the external environment into account, it is clear that a new review of bullying of staff in HEIs is timely.

A scoping review is appropriate where it is useful to identify the range and nature of the extant literature and to highlight research gaps and challenges [74]. It can act as a step to posing more specific research questions [75]. On this basis, a scoping review of workplace bullying in HEIs was undertaken with a view to mapping the literature, drawing conclusions about the overall research activity in this area, and proposing a research agenda going forward. This scoping review includes both bullying and sexual harassment on the grounds that both individual and organisational power are relevant in the context of changes in the sector in recent years, with the aim of exploring the volume, range, and nature of studies focused on workplace bullying in HEIs. The review further seeks to explore the unique context of HEIs and how this contributes to the enactment of and/or the response(s) to workplace bullying.

## 2. Methods

Drawing on the framework offered by Arksey and O’Malley (2005), this review will (1) identify research question(s), (2) identify relevant studies, (3) select appropriate studies, (4) chart the data, and (5) collate, summarise, and report results. An a priori protocol was not registered for this review. The PRISMA-ScR checklist [76] was used to guide good practice in the conduct of this review.

### 2.1. Research Questions

**Figure 1:**
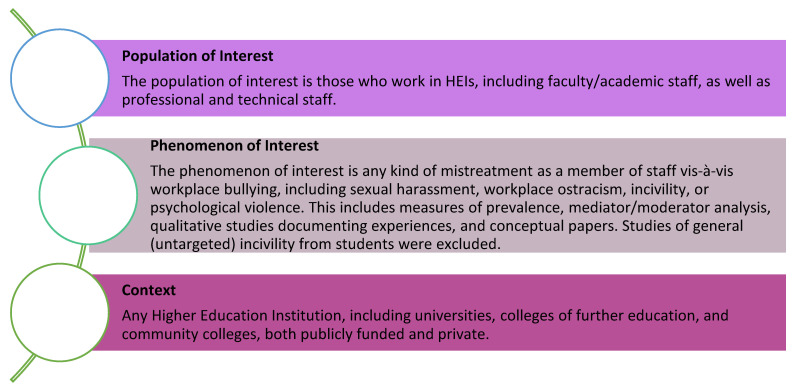
PPC framework for scoping review.

This review aims to identify the volume, range, nature, and characteristics of studies of workplace bullying in HEIs between 2003 and 2023. Furthermore, we seek to identify ‘*the extent to which the specific context of HEIs is considered to contribute to the enactment of and/or the response to workplace bullying*’. The PPC framework of phenomenon, context, and population was applied to structure the search strategy (see Figure 1). A wide range of terms were included to ensure the capture of various forms of bullying and harassment. We considered it important to explicitly include sexual harassment given the high levels in HEIs, recognising the argument that workplace bullying is just one face of institutionalised violence [77].

### 2.2. Identification of Relevant Studies

Search terms were identified from the literature and previous scoping or systematic reviews on various aspects of workplace bullying in different sectors (e.g., [78,79,80,81]) for the three blocks—phenomenon, context, and population—listed in Table 1 below. An online literature search (February 2023) was conducted using the following databases: Scopus (*n* = 229), PubMed (*n* = 343), Web of Science (*n* = 449), OVIDMedline (*n* = 170), Academic Search Complete (including PsychINFO (*n* = 1096), Cinahl complete (*n* = 194), ERIC (*n* = 280), and Business Source complete (*n* = 184)). Searches were run on abstract/title and abstract, as appropriate for each database, for the alternative terms for each block with the Boolean operator OR, and then blocks were combined with the Boolean operator AND. Limiters for language (English only), availability of the abstract, and publication dates (2003–2023) were applied.

### 2.3. Study Selection

**Figure 2:**
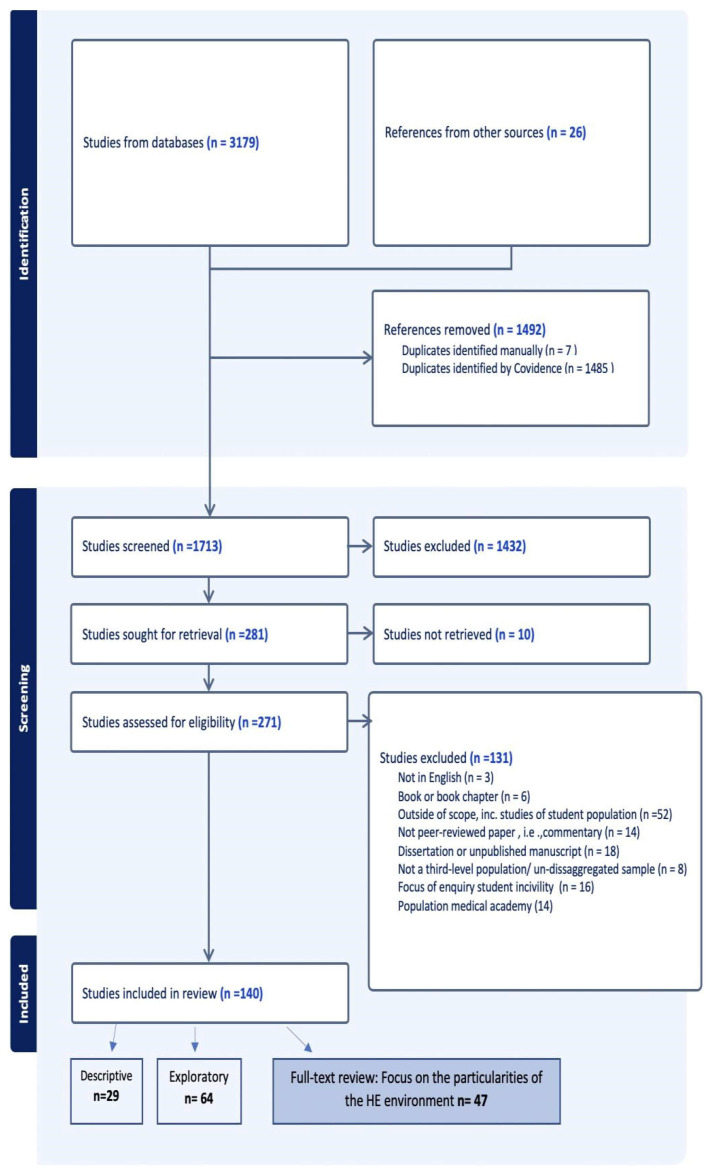
PRISMA chart (PRISMA (Preferred Reporting Items for Systematic reviews and Meta-Analyses) is a standard format for reporting systematic reviews).

Records were imported to COVIDENCE software (Covidence systematic review software, Veritas Health Innovation, Melbourne, Australia. Available at www.covidence.org.) (*n* = 3179) and de-duplicated, resulting in 1687 records for initial screening. References from other sources (*n* = 26) were added. Two reviewers (MH and RK) independently assessed titles and abstracts to identify studies relevant to the research question. Differences of opinion were resolved with discussion and examination of the full text where required. After screening by title and abstract, 281 records remained for eligibility assessment. Papers where the fieldwork was clearly set in universities or colleges of further education were included, while papers in which the fieldwork was undertaken in medical academies were excluded on the basis that they did not necessarily distinguish between the higher education environment and the clinical (teaching hospital) environment. Papers were excluded that were not in English, were not empirical studies, or were confined to the student population. Papers about bullying, harassment, ostracization, verbal abuse, or psychological violence within the staff of universities or colleges of further education were included, while those that addressed rising levels of incivility, rudeness, and entitlement from students towards staff were excluded, as this does not align with the definition of workplace bullying as ‘…*persistent and systematic victimisation of a colleague or a subordinate through the repeated use of various kinds of aggressive behaviours over a long* (taken to mean 3–6 months) *period of time and in a situation where victims* (while victim is term used in the quotation, target is the authors’ preferred term and will be used throughout the article) *have difficulty in defending themselves*.’ ([7], p. 41). However, on this basis, we deemed it appropriate to include papers that addressed the sexual harassment of a member of staff in their workplace by a student (see Figure 2). One hundred and forty papers were identified for charting purposes.

### 2.4. Data Charting

Scoping reviews require charting and collating the papers entered into the review [74]. In this regard, charting of the selected papers focused on key fields: date of publication, study aim, country in which data were collected, methods, type of HEI, population, and type of bullying. One hundred and forty papers were entered into the charting (or data extraction) stage to address the first research question.

As per the recommendations in [74,76], the summary data were charted (see Table 2). Charting also included the theoretical positioning of the paper. Papers that named a theoretical framework were identified. No judgment was made on the status of named theories, nor was any assessment conducted to establish whether named theories met criteria for theory evaluation [82]. A theoretical framework was ‘counted’ if described as a theory or theoretical framework by authors. In this way, 37 papers were found to identify a theoretical framework as a point of departure. These varied widely, including 26 theories or frameworks.

The full-text review stage of the review addressed the second research question, viz., to identify ‘*to what extent and in what way the specific context of HEIs is considered to contribute to the enactment of and/or the response to workplace bullying*’? In order to answer this, a second screening exercise was deemed necessary to select papers that gave consideration to contextual factors.

To this end, a further screening exercise was undertaken of the 140 charted papers that involved the categorisation of papers based upon the focus of enquiry within the individual paper. Consequently, papers were categorised based on whether (a) bullying was the focus of the paper with HEIs as an example of a workplace, for example, measuring the prevalence, presence of, or perceptions of policy, but with no reference to the specific context of an HEI; (b) there was a focus on general work environment factors such as organisational commitment, role clarity, etc., for example, exploring the work environment hypothesis [7], which posits that aspects of the psychosocial environment act as antecedents, mediators, or moderators for workplace bullying; or (c) the paper examined the specific context of HEIs as a workplace, i.e., aspects of the work environment, culture, or climate that are particular to HEIs. In this way, three categories of paper were created as follows:

1. Studies that are primarily descriptive, viz., descriptive reporting of levels of exposure with demographic or other (e.g., section or staff group) breakdown. The context is a workplace (*n* = 29).
2. Studies that are exploratory, i.e., aiming to advance the understanding of bullying and harassment through moderators, mediators, and/or processes and organisational nuances. The context is the workplace as a complex organisation (*n* = 64).
3. The focus on is on the particularities of the HE environment (beyond the brief mention of high levels of bullying/the topic being understudied, etc.), including power differentials in the context of gender inequity or students, and/or (b) the challenges inherent in the changing context of HE. Category three does not preclude category two. The categorisation is on the basis of the inclusion of contextual discussion within the paper (*n* = 47).

Studies in the third category (*n* = 47) were selected for full-text review. The papers were interrogated with the following question: *What are the contextual factors identified and discussed in this paper that contribute to or facilitate the enactment of and/or the response to bullying and/or harassment in the institution*? The research team divided these studies into groups, with each member undertaking full-text reviews, with quality cross-checking and discussion as needed. The 47 papers (see appendix) that identified and discussed contextual factors that contribute to or facilitate the enactment of, and/or the response to, bullying and/or harassment in HEIs were subjected to a thematic analysis.

## 3. Results

### 3.1. Collation and Summary of Data

With regard to the volume, range, nature, and characteristics of studies of workplace bullying in HEIs between 2003 and 2023 (see Table 2), the data charting process found diverse methodologies, with surveys predominating. Forty-two papers included a prevalence survey and thirty-two were qualitative studies. There were 25 mediator or moderator analyses, a greater number than cross-sectional analyses (*n* = 18), and only 1 study included an intervention. Most studies were undertaken in public universities or both public and private universities, and most were conducted on academic staff or all staff. Studies focusing on professional staff alone were relatively few in number (*n* = 16). Bullying, sexual harassment, and incivility were the main forms of ill treatment studied, and 91 studies were conducted in either the Americas, Oceania, or Europe. Of the 140 records, only 37 had a theoretical framework, although a further 30 discussed findings in the context of what can be broadly termed *power theory*. In 24 of these papers, the focus of enquiry was sexual harassment.

With regard to the specific context of HEIs contributing to the enactment of and/or the response to workplace bullying, academia was generally seen to be hazardous in respect to bullying: *“It seems that due to its history and special characteristics, academia is an arena that is highly vulnerable to bullying and inappropriate behaviour*.” ([83] p. 619). Academia is characterised by institutional structures built on hierarchies of gender, race, and class, and is evaluation-intense and competitive, valuing autonomy, expertise, and individualism in a way that makes it difficult to identify or label bullying in the traditional way. It also has de-centralised governance structures that make it difficult to dismiss staff [58].

### 3.2. The Impact of Neoliberalism

A strong theme that emerged in the papers was how the neoliberal economic model and the inevitable managerialist regime have aggravated these aspects of the working environment in a way that, in turn, greatly exacerbates the risk of bullying and of harassment, and reduces the likelihood of it being addressed. Relatedly, academia was seen to have complex, multi-layered, gendered power dynamics that provide multiple opportunities for abuses of power, in particular sexual harassment, but that also ‘switchback’ to maintain the power and privilege of a small group of people who are likely to be highly resistant to change.

#### 3.2.1. Neoliberalism Compounds Existing Contextual Factors

Almost half of the 47 papers (*n* = 22) discussed the malign influence of neoliberal values and their implementation in HEIs. Defining features of neoliberal universities such as *‘ever-intensifying workload, short-term contracts, job insecurity, funding pressures, excessive competitiveness, the power imbalance between managers and academics, and weakened union power (…)*’ along with a ‘…*constant preoccupation with finances, measurements, marketing and accountability*’ ([84], p. 199) are seen as commonplace, and staff have become resigned to them. The corporatisation of HEIs and the ‘*forced business model which is imposed to increase efficiency and ‘bottom line’ operating conditions*’ ([27], p. 281) are deeply implicated in the institutional processes that give way to bullying and harassment. Zwadzski and Jensen sum this up, stating that ‘*the neoliberalisation of academia has spurred normality in the form of management led practices in which bullying becomes part of the game*’ ([85], p. 406). These management practices go beyond traditional methods, combining management with an ideology that everything must be managed to increase efficiency, resulting in ‘*surveillance and micromanagement*’ ([86], p. 710) and ‘*a highly individualised, hyper-competitive, performance-driven ethos*’ ([87], p. 7). Specifically, the impact of neoliberal managerial practices was seen to greatly intensify workload stress and pressure: ‘*The profession has adopted new work patterns such as the metrification of academic outcomes, and the constant pressure to report on these*’ ([88], p. 127). In the context of increased job insecurity, precarity, and limited resources, it is easy to see how this is likely to lead to frustration and ultimately heighten the risk of aggression [54].

It was widely acknowledged that the academic environment has become intensely competitive and adversarial. Competition, once one element of academic life, now interlaces with every aspect of the work environment as staff compete for funding, titles, recognition, resources, and citations [89,90] in the pursuit of the chimera of ‘excellence’. This ignores the fact that, as excellence is relative, most will not attain it, and being gendered, many will find it harder, even impossible, to prevail [86]. Metrification and performativity are seen to have changed the academic labour process. Staff are now heavily dependent on the evaluation of their peers for promotion or academic management roles [91], which have proliferated in the service of managerialism. Such evaluations, claiming meritocratic processes, employ performance criteria that involve ‘*subjective, often ambiguous, criteria, as evident in reviews of scholarly/intellectual contributions, department- and college-wide service, continuing growth, and community service. Few institutions have clear standards for judging such contributions and, instead, rely on general guidelines or descriptive criteria (…) Such judgments often lead to perceptions of distributive injustice, unfair treatment associated with outcomes and procedural injustice, and unfair treatment associated with the decision-making*’ ([54], p. 9), and are greatly intensified in highly competitive neoliberal universities. Zawadzki, for example, points out that the much-vaunted meritocracy is simply a ‘*a tool for neoliberal reforms to legitimize further injustice*’ ([85], p. 399). These processes undermine professional competence [92], creating significant professional vulnerability [93]. Competition produces ‘winners’, which justifies it in the managerialist mindset, yet inevitably there are always many more losers. This in turn fosters envy, bitterness, and resentment, conditions which cannot but raise the risk of negative behaviour such as incivility, sabotage, or predation.

#### 3.2.2. Precarity and Job Insecurity

In the context of precarity, job insecurity, assessments for awards, and other indicators of prestige that determine advancement, the potential for both bullying and the silencing of voices is significantly raised [87]. The power gap inherent in precarity increases the likelihood that bullying or harassment will occur and that it will not be reported.

The articulation and acceptance of the robust critique of ideas is acknowledged as an essential aspect of academic life, but one where the managerial, monetised environment driven by neoliberal values has raised the stakes considerably for ‘winners’ in the game of metrics and prestige indicators. This was seen to contribute to an increasingly harsh and punitive climate, where person-related belittlement and professional undermining are commonplace [94], incivility is tacitly accepted, assessment can be weaponised, fear can be employed in a way that can easily segue into bullying, and where ‘*demonstrations of power are seen as reasonable and warranted if an individual is to succeed*’ ([90], p. 116). Indeed, a push against anti-incivility policies was identified in the interests of open criticism and the name of academic freedom [95]. In this view, the critique of staff in the service of excellence and performativity should be permitted, even if uncivil. They observe a deliberate fuzzing of the boundaries between the vigorous criticism of output, intellectual work, or theoretical propositions, and abrasive behaviour, mockery, and humiliation—a concerning development in the US context [95].

Critique of the system in neoliberal universities, as opposed to the work and output of individuals, is not safe, and this in turn increases conformity and reduces the likelihood of exposing bullying when it occurs. Several papers noted the requirement in the corporate model for conformity and the potential silencing of voice. The insecurity and competitive tension in neoliberalised HEIs are such that conformity and non-confrontation are the only safe option if one wishes to progress; Berquist et al. found that ‘*expressing opinions which were outside of the dominant discourse carried risks. Some even stated that they would not survive if they displayed any form of resistance to the dominant discourse. Participants spoke about keeping their heads down and not rocking the boat*.’ ([90], p. 116). Staff conform to bullying regimes to survive. Similarly, Zabrodska et al. find that ‘*the fear of being cast out, and of not being recognized (…) prevented the narrator from engaging in overt forms of resistance. In order to remain viable academic subjects, they had to remain silent, at least for that moment*…’ ([86], p. 717). While neoliberalism insists on the need for strong managerial control, there are blurred lines between this type of management and bullying, and such bullying *‘can very easily be concealed*’ ([86], p. 710). The structures within HEIs which involve autonomous units, where increasingly responsibility for management and governance is being devolved to academic schools or departments, allows bullying to remain hidden within a unit, and bullies to become the arbitrators of their own bullying actions [96]. Relatedly, O’Connor et al. draw attention to a less recognised impact of managerialism: the shift within HR from being a body concerned with the wellbeing of personnel to that of a corporate apologist concerned with presenting the institution in the best possible light (p. 8 citing [62]). The institutional branding that is a feature of neoliberal universities allows the prioritization of reputation over the health and welfare of staff [97].

It was also acknowledged by a small number of researchers that these processes, in addition to making individual or interpersonal bullying and harassment more likely, in and of themselves, constituted institutional bullying (e.g., [98,99]). In this way, the impact of neoliberalism has been to make the enactment of bullying more likely and the possibility of reporting and seeking redress less likely. The economic imperatives of corporatisation effectively override moral or ethical considerations.

### 3.3. Complex and Malevolent Gendered Power Dynamics

It was widely acknowledged that HEIs are markedly inequitable. Despite being sites of study that understand social inequity, institutional structures reflect hierarchies of gender, race, and class [100], with power unequally distributed both horizontally and vertically. HEIs are male-dominated spaces where the privileged masculinity of power is embedded and normalised in a way that enables abuses of power, as articulated by O’Connor et al.: …‘*those who are most likely to be victims are those who are in structurally unbalanced relationships in terms of power, and/or culturally defined as ‘Other’, e.g., women, especially postdocs and postgraduates or first-year undergraduate students, racial/ethnic minorities and other culturally vulnerable groups who are multiply devalued in terms of race, class, gender, sexuality, etc*.’ (p. 3). Herein lies a very significant potential for the abuse of power that is unique to HEIs.

Power relations extend beyond the traditional hierarchical power found in other work environments [84,93,101], including normalised dependencies between undergraduates and academic staff in the context of assessment, or as mentors tasked with guiding students and junior scholars, and between postgraduate students, who may also be employees, and supervisors, who may also be PIs, a situation brought into sharp focus by precarity. In addition to the normal dependencies between early-career staff and established or tenured staff, both precarity and hyper-competitiveness intensify dependencies that exist in terms of the need to ‘pay forward’ for promotion or inclusion in publications or in research projects [87]. As such, students and junior and/or precariously employed staff may find themselves in particularly vulnerable positions if they experience bullying or harassment.

With regard to student dependencies, extreme cases were discussed where an organisational culture characterized by patriarchal dynamics intersects with the national culture or with the economic climate. In China and Benin, for example, senior staff have considerable power and intellectual influence, including power over entry into the doctoral programme and job opportunities in wider society, facilitating the enactment of exploitation and sexual harassment [102,103]. The sexual harassment of female graduate students in the form of transactional sex, the most serious manifestation of sexual abuse, is tolerated, normalized, and condoned by staff and students alike. Eller maintains that ‘*transactional sex in a campus setting must be grounded in an understanding of the numerous intersecting inequalities that encourage these relationships. Heteronormative gender dynamics exert pressure on women to acquiesce to men in positions of authority, and authority over grades is further reinforced by a professor’s age and educational status*’ ([103], p. 754). A traditional model of masculinity as authoritative and femininity as submissive prevails, and appears to be unchallenged, intensified by the intersection of expert and positional power.

In this way, HEIs offer many sites for potential power abuse. The concept of gendered organisations was evident in discourses of sexual harassment, where culture, structures, assumptions, and discursive practice are essentially masculinist processes that permeate all aspects of the work environment [98,101,104,105,106]. Guizardi et al. describe this as the androcentric and patriarchal nature of academia where men define what matters, what is normal, what is valued, and what ‘counts’—ways of working that inherently disadvantage women in respect to tenure, promotion, and advancement, and that are identifiable as subtle forms of institutional violence. Power is not only exercised in overt aggressive acts, it is exercised in subtle ways—‘*The slippery nature of the forms of violence experienced by women in academic environments*’ ([104], p. 50)—including insults, put downs, explicit low expectations, patronising comments, non-recognition of women’s achievements, heavier teaching, and lower pay. Lombardo et al. similarly describe this as the normalisation of violence: “*Sexual harassment occurs in institutionally gendered university settings that are not only shaped by formal rules that are consciously designed and written, but also informal rules, the unwritten norms and practices that are rarely acknowledged by institutional actors despite having collective effects*” ([106], p. 9). The persistence of these everyday acts, not identifiable individually as harassment or abuse and enacted by women as well as men, collectively serve to reproduce male domination [104]. This leads to a ‘*blindness within the academic community to sexual/sexist harassment as a problem, revealing high levels of normalization of daily harassment practices*’ ([106], p. 20).

As such, sexual harassment was seen to be both agentic [107] and malevolent [97]: an exercise of power to maintain the situation of vulnerability and subordination of those who are outside the core power structures of the organisation, typically those who do not display hegemonic masculinity. In its most sinister conceptualisation, sexual harassment is seen to maintain and strengthen male power in universities. It is part of the apparatus of hegemonic masculinity, defined as ‘*a cultural norm that endorses the traditional power of men and restrains women from attaining and maintaining leadership positions*’ ([107], p. 2). Sexual harassment is not just something that happens to certain women, but it is a tool that is used to maintain the status quo, which in academia is a male-dominated management structure. Men hold the majority of top posts and therefore are strongly invested in maintaining this situation. Understanding harassment, and by extension bullying, in HEIs requires an understanding of the operation of power, especially hidden or stealth power [86].

While neoliberalism does not necessarily create such toxic masculinities, it both reproduces and aggravates them. Phipps, for example, argues that in the drive to monetise everything, an economic-based ‘reckoning’ occurs, where some individuals (for example, those bringing in large amounts of research funding) are deemed to be of greater value and therefore will be protected from investigation, and where the institution will be protected over individuals, both contributing to a silencing and submerging of bullying or harassment claims: …‘*our value is not inherent but defined by what we bring to the institution, which can be used or exchanged (…) power/value relations, which both reflect and perpetuate other relations such as gender, race and class*’ resulting in an ‘*unwillingness to tackle people who are these so- called research superstars and the way they behave, for fear that they may leave and take their money and their publications with them*.’ ([97], p. 234).

## 4. Discussion

This scoping review aimed to explore what is known about workplace bullying and harassment in HEIs and to map the factors that are particular to HEI workplaces. The review identified the volume, range, nature, and characteristics of studies of workplace bullying and harassment in HEIs between 2003 and 2023. The review identified 140 records that explored bullying and harassment in HEIs, including a wide range of methodologies. Forty-seven papers were selected for full-text review to explore how the specific context of HEIs contributed to the enactment of and/or the response to workplace bullying and harassment.

There has been a steady output of work since the publication of Henning’s systematic review. Henning et al. reviewed 51 studies, following a search for publications over a 20-year period (1994 to 2013) [71]. The present study, also over a 20-year period (2003–2023), entered 140 studies into the review for charting, resulting in a subset of 47 for full-text review. Of the 140 records, only 37 had a theoretical framework as a point of departure, although a further 30 papers discussed findings in the context of *power theory*, and in particular *gendered power* in organisations. For most, but not all (*n* = 24), of these papers, the focus of enquiry was sexual harassment.

The data charting of the 140 records found diverse methodologies, with surveys predominating (*n* = 42). Thirty-two studies were qualitative in nature. The latter is a significant increase on Henning et al.’s review, which found only five qualitative studies [70], representing an increase in interest in the lived experience of bullying and harassment in HEIs and an acknowledgment of the importance of targets’ perspectives. There were 25 mediator or moderator analyses reflecting an understanding of the need to explore pathways and mechanisms beyond simple associations, as recommended by Nielsen and Einarsen (2018) [1]. Of particular note, however, is the fact that only one study included an intervention, highlighting an important gap in the research and signposting a priority for future research. Most studies were undertaken in public universities or both public and private universities, and most were conducted on academic staff or all staff. Studies focusing on professional staff alone were relatively few in number (*n* = 16), exposing another research gap. Ninety-one studies were conducted in either the Americas, Oceania, or Europe, indicating a strong bias to Western models of industrialisation. This reinforces calls for an enhanced global approach including culture-specific studies that acknowledge workforce composition and cultural differences in workplace relations (e.g., [108,109]).

The full-text reviews undertaken here, consistent with the wider literature, confirm the existence of particularities or contextual factors contributing to bullying and harassment in HEIs, with a very limited discussion of individual factors. Context, however, is increasingly recognised as essential for a true understanding of bullying in workplaces [108,110]. More generally, improving health in settings where there is close attention to context increases the likelihood of success, as interventions can be optimised for specific contextual contingencies [111].

The neoliberal ideology that has swept through the HEI sector, bringing in its wake governance approaches that include managerialism and marketisation implemented through performativity, hyper-competitiveness, and precarity, has been subjected to significant criticism in recent years. Neoliberalism has been associated with challenges to integrity and academic freedom [64], de-professionalisation [112], rising student debt [113], and a climate of detrimental change in academia [114]. To this corpus of deleterious effects we add bullying, harassment, and institutional violence. Drawing on an ecological framework [86], the neoliberal ethos effects bullying at three levels. At the *micro*, interpersonal–relational level, the conditions for frustration leading to aggression are a daily occurrence [53]. At the *meso* or organisational level, the structures and processes of performativity and competitiveness, combined with precarity, create situations where reporting or calling out bullying and harassment are effectively inhibited. At a *macro* level, this amounts to a form of institutional violence [76], polarising power relations and driving a culture of threat and fear. Hitherto under-researched aspects of neoliberalism, such as the potential for bullying and harassment in the context of the dependence of HEIs on external donations, have yet to be explored.

An extensive amount of scholarly literature is dedicated to the under-representation of women in senior positions in higher education. Understanding the concept of gendered organisations is required to understand how this intersects with neoliberalism and how it facilitates institutional violence. The embracing of neoliberal ideals which valorise competition, brutal evaluations, league table winners, and research ‘stars’ is a heteronormative masculinised perspective. Bullying and harassment are embedded in gendered neoliberalised HEIs, not through benign neglect, but as ways of ensuring that the power structures that favour traditional patriarchal masculinity are preserved and reproduced. The findings here are consistent with Berlingeri’s analysis of power as an exercise of institutional violence [77] and Agocs’ work on institutional resistance to gender equality [115].

There are aspects of power that are unique to the higher education setting. In addition to hierarchical power (seniority), power based on expertise also attaches to academic rank. In the situation where managerial posts are appointed based on academic expertise, seniority and rank, and typically gender, an extraordinary nexus of power exists, wherein junior staff are highly vulnerable. The impact of this power accumulation overwhelms the organisation’s boundaries, making it very difficult to be challenged in any context. Power and its exercise are central to the understanding of bullying and harassment, not only in the context of interpersonal relations but at the level of the institution. The ‘power and politics’ context of bullying and harassment in HEIs has to be acknowledged. Power needs to be exercised in work organisations, but appropriate protections need to be in place to prevent the abuse of power, as acknowledged by Mary Parker Follett in her statement “*I do not think we shall ever get rid of ‘power-over’; I do think we should try to reduce it*.” [116], consistent with the recognition that addressing workplace bullying and harassment requires a sociological as well as psychological perspective [117].

**Figure 3:**
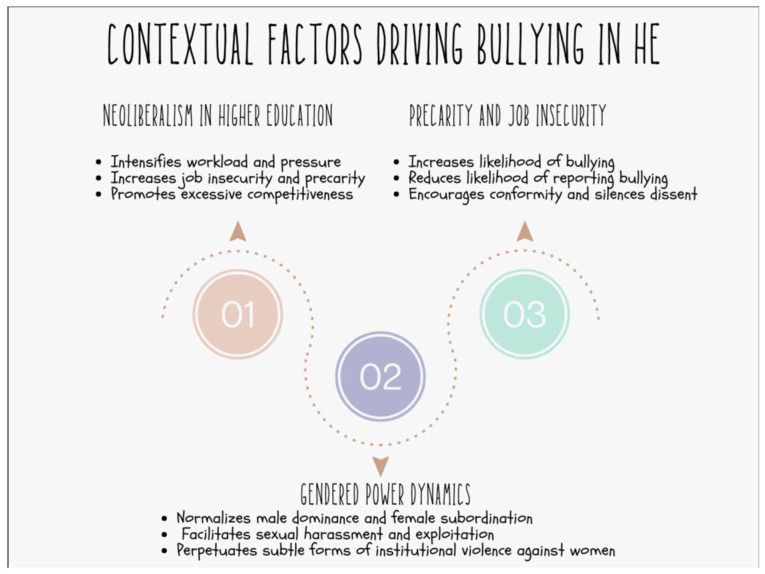
Contextual factors driving bullying in higher education.

As if there were not already a host of other reasons for turning back the tide on neoliberal governance, this review crystallises the importance of addressing bullying and harassment at the highest possible level and helps explain why individualised case management approaches [58] do not work and will not work in higher education. We summarise these factors in model form in Figure 3 below.

## 5. Conclusions and Future Directions

Limitations of scoping reviews notwithstanding (e.g., the application of limiters, time delay between searches and publication, etc.), this scoping review demonstrates that research on workplace bullying and harassment in higher education continues to advance, yet also exposes research gaps. The majority of the work to date has been conducted in industrialized nations with associated cultural lenses. Only 15 of 140 studies were conducted in Africa, for example. Extending research into emerging economies and non-Westernized cultures will better reveal the intersection between the various biases that attend the problem. Global perspectives may also be illuminating. Additionally, research to date has been over-focused on academic staff, and ongoing study should include professional staff, whom are also affected by the contextual factors identified here. The literature would benefit from greater attention to exploring issues from a theoretical standpoint. Despite the persistence of surveys to measure prevalence, it is evident from this scoping review that the priorities for future research lie in embracing increasingly nuanced interdisciplinary paradigms. Such studies need to move beyond simple prevalence estimates to address the pernicious effects of neoliberal governance models and the complex and systemic exercise of multi-layered power dynamics that extend throughout and beyond the boundaries of organizations. Finally, intervention research is clearly needed, but will require close attention to particularity, culture, and contextual factors within HEIs.

## Figures and Tables

**Table 1:** Search terms and Boolean operators.

**Phenomenon**	**AND**	**Population**	**AND**	**Context**
“Sexual Harassment”		Faculty		“College of further Education”
OR		OR		OR
“Psychological aggression”		Lecturer*		“Higher Education institution”
OR		OR		OR
“Psychological violence”		Academic*		University
OR		OR		OR
Bully*		Professor*		“Tertiary education institution”
OR		OR		OR
Incivility		Staff		College
OR				
Mistreatment				
OR				
Ostracism				

**Table 2:** Summary of charted data.

Methods Employed	Number
Prevalence (survey)	42
Intervention (e.g., before vs. after, or (q) experimental design)	1
X-sectional (e.g., correlational)	18
Mechanism, i.e., mediator/moderator analysis	25
Factor analysis	10
Outcome quantitatively measured (e.g., effects as measured by a scale)	22
Qualitative study (e.g., focus groups or interviews) *	32
Economic evaluation	1
Review (narrative, systematic, or scoping review)	5
Conceptual	11
Perceptions or attitudes towards events or policy	21
Documentary	6
Mixed methods (combined qualitative * and quantitative)	5
Testing or developing a model	5
Other ^§^	7
**Focus of Enquiry: Type of Institution**	
Public university	74
Private university	6
University (type unstated)	17
College (e.g., community, nursing, etc.)	6
Mixed	26
Unclear	4
Not applicable	7
**Focus of Enquiry: Population**	
Faculty/academic	76
Combined students and staff	22
Professional staff (administration, library, laboratory, technical)	16
All staff (i.e., not specified)	24
Other ±	2
Students (as perpetrators sexual harassment)	2
**Focus of Enquiry: Types of Ill Treatment**	
Workplace bullying	62
Sexual harassment	49
Gender-based violence	8
Incivility	31
Institutional bullying	1
Workplace ostracism	4
Mobbing (i.e., group bullying)	4
Toxic leadership	3
Gender-based discrimination	4
Unclear	1
**Focus of Enquiry: Continent and Country ^$^**	
America (USA: 38; South America: 3; Canada: 4)	45
Europe (UK: 10; Estonia: 5; Sweden: 5; Finland: 3; Ireland: 4; Spain: 3; Czech Republic: 2; Italy: 2; Albania, Croatia, Cyprus, Denmark, Lithuania, Norway: 1 each)	40
Asia (Pakistan: 7; Turkey: 7; China: 4; Malaysia: 2; Indonesia: 2; India: 2; Jordan: 2; Japan: 1; Singapore: 1; Iran: 1)	29
Africa (South Africa: 5; Botswana: 2; Nigeria: 2; Ethiopia: 2; Benin, Egypt, Zambia, and Zimbabwe: 1 each)	15
Oceania (Australia: 5; New Zealand: 1)	6
**Dates of Publication**	
2003–2008	11
2009–2013	17
2014–2018	34
2019–2023	74

The ‘count’ for fields exceeds 140, as studies often employed more than one method, measured more than one type of ill treatment, were cross cultural, etc. * An open-ended question contained within a survey was not counted as a qualitative method for the purposes of the review. ^§^ Netography, ethnography, autoethnography, collective biography, experiment, case study [*n* = 2]. ± Graduate workers; ^$^ country as named in article.

## Data Availability

No new data were created or analyzed in this study.
